# Self-assembled nanoparticles of costunolide and glycyrrhizic acid for enhanced ulcerative colitis treatment

**DOI:** 10.1186/s12876-024-03313-9

**Published:** 2024-07-11

**Authors:** Hao Fu, Xiao Zheng, Ke Xu, Yuge Zhang, Mengxia Wu, Min Xu

**Affiliations:** Pharmacy College, Chengdu University of Traditional Chinese Medicine, Chengdu, China

**Keywords:** Ulcerative colitis, Costunolide, Glycyrrhizic acid, Self-assembled nanoparticles, Anti-inflammatory, Antioxidant

## Abstract

Ulcerative colitis (UC) is a persistent inflammatory condition that specifically targets the colon and rectum. Existing therapies fail to adequately address the clinical requirements of people suffering from this ailment. Despite the acknowledged potential of nanomedicines in the field of anti-inflammatory treatment, their widespread use in clinical settings is impeded by their expensive nature and the uncertainty surrounding their safety profiles. This study illustrates that two naturally occurring phytochemicals, Costunolide (COS) and Glycyrrhizic acid (GA), form carrier-free, multifunctional spherical nanoparticles (NPs) through noncovalent interactions, such as π–π stacking and hydrogen bonding. The COS-GA NPs displayed a synergistic anti-inflammatory effect, providing much more evidently improved therapeutic benefits for dextran sodium sulfate (DSS)-induced UC mice due to more effective reduction in inflammation and oxidative stress than did equal dosages of COS or GA used alone. In addition, COS-GA NPs have biocompatibility and biosafety properties unique to them. This study will serve as affirmation of the potential of COS-GA NPs as innovative natural anti-inflammatory and antioxidant activities and also such agents as drug discovery in UC, leading possibly to better outcomes in people living with this disabling condition.

**Supplementary Information:**

The online version contains supplementary material available at 10.1186/s12876-024-03313-9.

## Introduction

Ulcerative colitis (UC) is a chronic, inflammatory state manifesting as bloody diarrhea, usually starting in the rectum and eventually spreading to involve most parts of the colon [1]. The increasing prevalence of ulcerative colitis now poses a public health challenge. Treatment options for UC include aminosalicylates, corticosteroids, immunosuppressive drugs, and monoclonal antibodies directed against tumor necrosis factor-α (TNF-α). Although these treatments have improved the quality of life of patients, their effectiveness is partial [2]. Moreover, the presence of adverse effects such as tremors and sleeplessness restricts their prolonged utilization.

Natural medications present a hopeful substitute since they are reasonably priced, easily obtainable, and safe. Nevertheless, their efficacy is sometimes hindered by inadequate solubility, bioavailability, and stability [3]. Hence, this biological effectiveness of these natural constituents needs to be enhanced through the drug development procedures to allow for their clinical use. Despite the extensive research conducted on drug delivery methods [4], classical nanomedicines have not been able to reach clinical requirements due to their intricate production and restricted human biosafety [5]. A novel strategy to circumvent these obstacles is self-assembly, an inherent mechanism in which molecules spontaneously arrange themselves into nanoparticles (NPs) under appropriate circumstances, resulting in enhanced physical and biological characteristics [6]. The utilization of self-assembly is widespread in the manufacturing of nanomedicines. Although spontaneously self-assembled NPs with potential anti-UC properties have several uses, there is a lack of reports on them. Our research aims to create innovative NPs without carriers for the treatment of UC, building upon previous findings.

Costunolide (COS), a principal sesquiterpene lactone, was initially isolated from *Radix Aucklandiae*, a significant component of traditional Chinese medicine for treating various gastrointestinal disorders [7]. Studies have demonstrated that COS alleviates symptoms of UC in mice [8]. Glycyrrhizic acid (GA), a pentacyclic triterpene and saponin derived from Uralic glycyrrhizae, possesses anti-inflammatory, antiviral, and other pharmaceutical properties [9, 10]. Research confirms GA’s therapeutic effects on UC [11, 12]. There are more reports on GA self-assembly studies and fewer on COS [13, 14], but COS has the potential for self-assembly [15]. More importantly, there are no studies on GA and COS self-assembly.

**Fig. 1:**
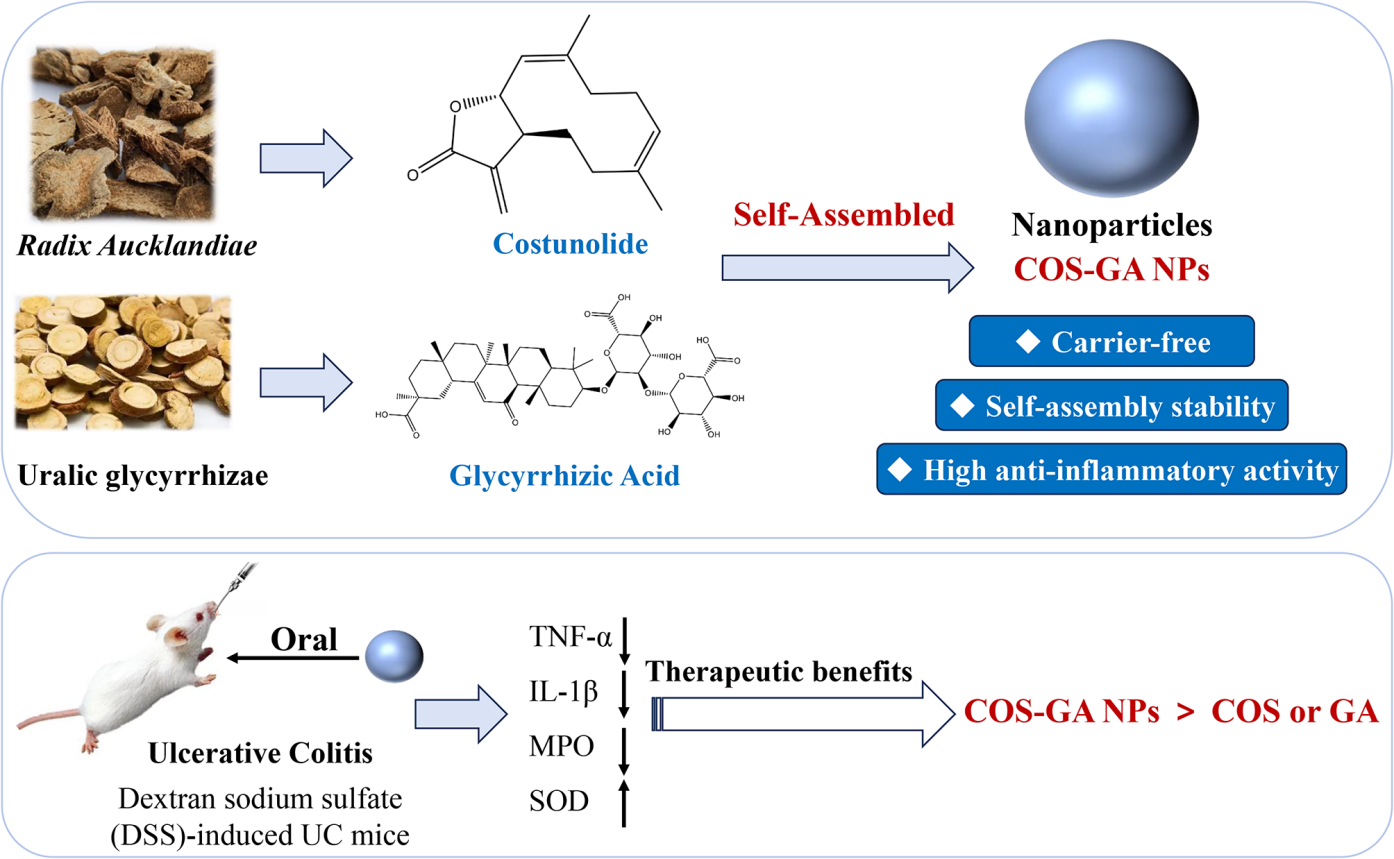
Schematic illustration of COS-GA NPs and their treatment effects on ulcerative colitis in mice

This research introduces COS-GA NPs, a new class of carrier-free NPs made by capitalizing on the chemical and physical characteristics of GA and COS through a conventional precipitation technique. The anti-inflammatory activities of COS-GA NPs are superior to those of COS and GA molecules alone. In addition, polymeric NPs have demonstrated promising therapeutic effects in the treatment of UC in mice that is induced by dextran sodium sulfate (DSS). Due to its high degree of similarity to human reported clinical symptoms and histological features, this mouse model sees extensive application. A novel perspective on binary carrier-free NPs is presented in this study (Fig. 1).

## Methods

### Preparation of COS-GA NPs

COS (NO. CHB231026) and GA (NO. CHB231107) were obtained from Chengdu Chroma-Biotechnology Co., Ltd. (Chengdu, China). COS and GA were dissolved in tetrahydrofuran (THF) to obtain a solution with a concentration of 10 mM, respectively. The two solutions are then mixed and stirred at 35 °C for 30 min and then centrifuged at 8000 rpm to obtain the mixture. Afterwards, a 2 mL mixture containing COS and GA was slowly introduced into 50 mL of ultrapure water and agitated continuously for 90 min at a temperature of 25 °C, in order to facilitate the evaporation of THF. Finally, the COS-GA NPs were synthesized.

### TEM analysis of COS-GA NPs

The morphology of the COS-GA NPs was determined using a transmission electron microscope (TEM) with a JEM-1400plus made in Tokyo, Japan. A 0.1 mL aliquot of the COS-GA NPs was dropped onto a copper mesh and covered with 2% aqueous phosphotungstic acid for 10 min.

### Particle size, PDI and Zeta potential of COS-GA NPs

COS-GA NPs particle size, polydispersity index (PDI), and zeta potential were measured by dynamic light scattering (DLS) method using Malvern Zetasizer Nano-ZS (Zetasizer Nano ZS 90, Malvern Instruments, UK). The size and PDI of COS-GA NPs were determined from nanoparticle suspension of appropriate amount of the drug loaded nanoparticles dispersed in water in a clear disposable sizing cuvette. Zeta potential was analyzed by a clear zeta cell.

### Molecular dynamic simulation

Ten molecules of COS and GA were combined and solvated in a cubic box of 10 × 10 × 10 nm; as per the usual rules of periodic boundary conditions, the resultant is taken to be the simulation system [3]. The simulation software uses Gromacs2022.4, the force field is GAFF, the water molecule model is TIP3P, the water box size is 10 × 10 × 10 nm, and 50 COS molecules and 50 GA molecules are randomly filled. A two-step energy minimization is performed for the initial simulated system, the first step is done 10,000 times using the most rapid descent method (STEP), and the second step is done 5,000 times using conjugate gradient (CG). After energy optimization NPT simulation is done for 200 ps, after NPT simulation finished simulation is done for 100 ns, temperature control algorithm is done by V-rescale, pressure control algorithm is done by parrinello-rahman, simulation step is 2 fs, temperature is 300 K. Electrostatic interactions are done by ionic lattice (PME) algorithm to compute the Coulombic interactions, electrostatic and van der Waals interactions with a truncation radius of 10 Å. Hydrogen bonding was constrained using the LINCS algorithm, with conformations saved every 10 ps, and visualization of the simulation results was done using the Gromacs embedded program and Pymol.

### Determination of COS-GA NPs stability

Stability of COS-GA NPs was also tested under severe conditions of thermodynamic and gravitational stability. Gravitational stability was tested in terms of centrifugation forces at 6000 and 12,000 rpm for 30 min each. It was checked for thermodynamic stability by exposing to cycles of 20 °C and − 20 °C, three times, and keeping at 50 °C and at 4 °C for 30 min each. Subsequently, particle size and ZP were measured.

### DSS-induced UC mice model establishment

SPF (Beijing) Biotechnology Co., Ltd., located in Beijing, China, provided a total of thirty healthy male ICR (Institute of Cancer Research) mice, with an age range of 6 to 8 weeks. The experiments were approved by the Medical and Experimental Animal Ethics Committee at CDUTCM under Ethics Approval No. CDUTCM-2023-129, in accordance with established ethical norms.

The mice were subjected to a 7-day adjustment period in a controlled environment (temperature maintained at 25 ± 1 °C, relative humidity ranging from 40 to 70%, 12-h light cycle), with sufficient water and food provided. UC was induced by administering DSS for a period of seven consecutive days. The 36 mice were randomly divided into model group (*n* = 30) and control group (*n* = 6). The control group of mice maintained a conventional diet, whereas the model group was administered a 3.0% w/v DSS solution (NO. SHE230514, Seebio, Shanghai, China) for 3 days. At the end of the modeling on day 3, the model was evaluated according to the DAI score, and a DAI score > 1 was considered a successful modeling [16]. All 30 mice were successfully modeled and then randomly divided into DSS, COS, GA, COS-GA, and 5-ASA (5-aminosalicylic acid) groups. From Day 4 to Day 10, the COS, GA, and COS-GA groups (the drugs suspended in 0.5% CMC-Na) received a daily oral dose of 10 mg/kg. The group that tested positive for 5-ASA (5-ASA suspended in 0.5% CMC-Na, NO. SX230122, ShanghaiYuanye, Shanghai, China) got a daily dose of 400 mg/kg of 5-ASA for 7 days. The colitis severity was assessed and measured on a daily basis using the Disease Activity Index (DAI). After referring to The ARRIVE guidelines 2. 0 (https://arriveguidelines.org/arrive-guidelines), mice were killed by cervical dislocation.

### Histological staining

Following a thorough washing with PBS, the mice’s colons were carefully removed and stored for 24 h in a solution containing 4% paraformaldehyde. Afterwards, 3 μm thick slices were cut from the tissues and placed in paraffin. The sections underwent deparaffinization and were then stained for histological examination using a Hematoxylin and Eosin (H&E) staining kit.

### ELISA test of cytokines

The concentrations of tumor necrosis factor-alpha (TNF-α, Catalog No. HF20231222), interleukin-1β (IL-1β, Catalog No. HF20231220), myeloperoxidase (MPO, Catalog No. HF20231228), superoxide dismutase (SOD, Catalog No. HF20231229) in colon tissues were determined using Enzyme-linked immunosorbent assays (ELISA) kits supplied by HeFei BoMei Biotechnology Co., Ltd., Hefei, China.

### Biochemical detection of liver and kidney functions

The whole blood was centrifuged at 12000r/min for 10 min to extract serum. After that, the liquid part was frozen at -80 °C for later use. The biochemical laboratory department at Chengdu University of Traditional Chinese Medicine assessed the kidney and liver’s biochemical function.

### Statistical analysis

After running the data through SPSS (version 22.0), the results were presented as the mean value with or without the standard deviation. An independent Student’s t-test with a two-tailed method was used to compare the two groups. Conversely, one-way ANOVA followed by the Tukey post hoc test was used for multiple group comparisons. Furthermore, non-normal data were analyzed using the Kruskal-Wallis H test. To establish statistical significance in this study, a p-value less than 0.05 was utilized.

## Results

### Preparation and morphological characterization of COS-GA NPs

**Fig. 2:**
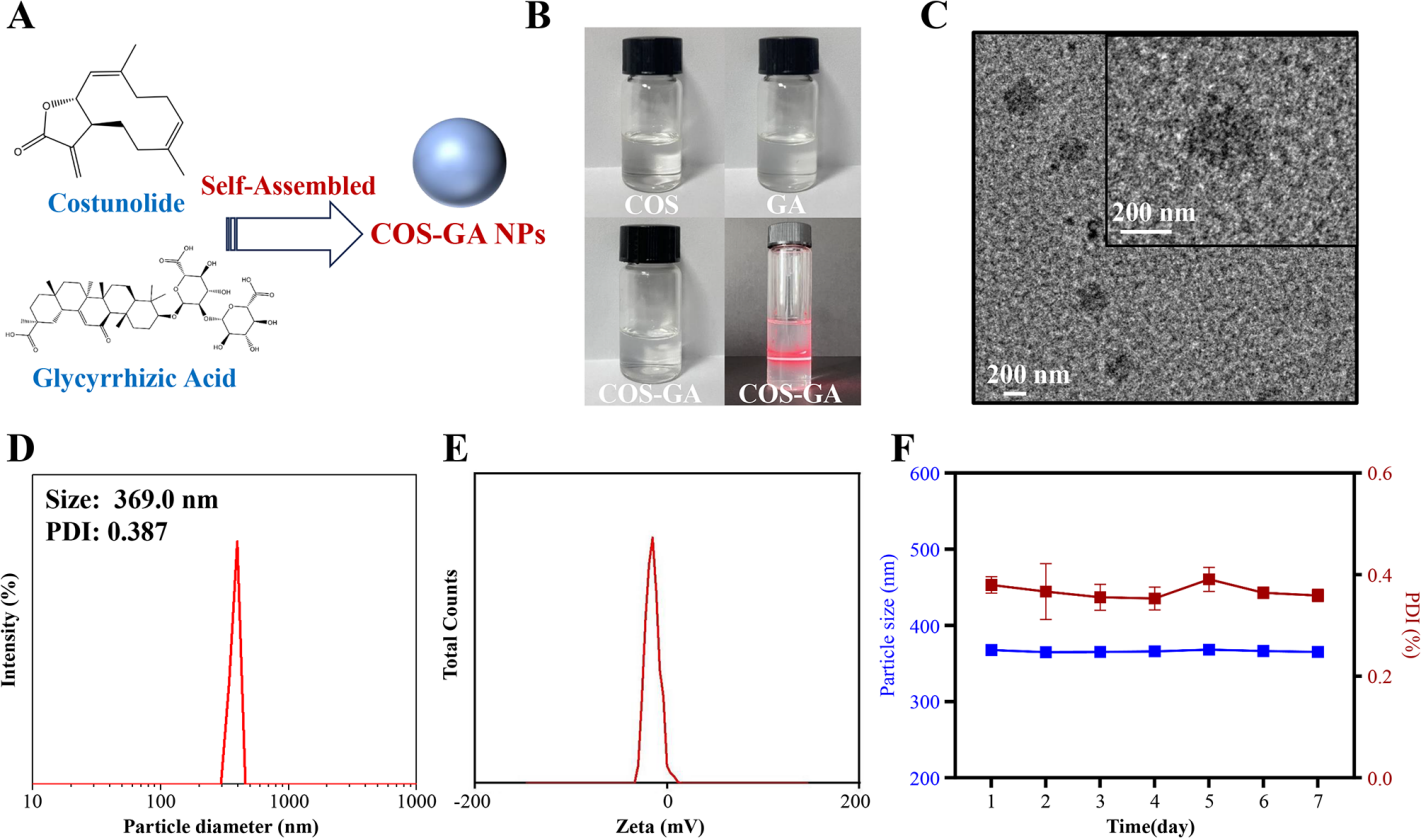
Analysis of COS-GA NPs’ physical characteristics and morphology. (**A**) A schematic showing how COS-GA NPs are made and tested. (**B**) Pictures showing COS, GA, and COS-GA NPs in liquid solutions. (**C**) A set of pictures captured with a scanning electron microscope. (**D**) Results of dynamic light scattering and distribution of size. (**E**) The zeta potential of COS-GA nanoparticles. (**F**) The COS-GA nanoparticles in complete culture media were studied for 7 days, the red line indicates the change in COS-GA NPs ‘s PDI over time, and the blue line indicates the change in COS-GA NPs ‘s size over time

COS-GA NPs were created by combining COS and GA aqueous solutions using a standard nanoprecipitation technique (Fig. 2A). Figure 2B shows that there was a noticeable Tyndall effect in the COS-GA NPs, even though the COS and GA solutions separately were plainly identifiable. Figure 2C shows the results of TEM, which revealed that the COS-GA NPs had a spherical form and a diameter ranging from 200 to 300 nm. The measurement results showed that the average diameter of the COS-GA NPs was 369.0 nm. Figure 2D shows that these NPs had a polydispersity index (PDI) of 0.387, which means that they were highly dispersed in water and were able to effectively penetrate gastrointestinal epithelial cells. The zeta potential (ZP) of the COS-GA NPs was determined to be -12.0 mV, as shown in Fig. 2E. Furthermore, it was discovered that the particles’ size and the PDI remained constant, suggesting that the COS-GA NPs exhibit considerable stability in the entire culture media for a duration of 7 days (Fig. 2F).

### Self-assembly molecular dynamics simulation of COS-GA NPs

**Fig. 3:**
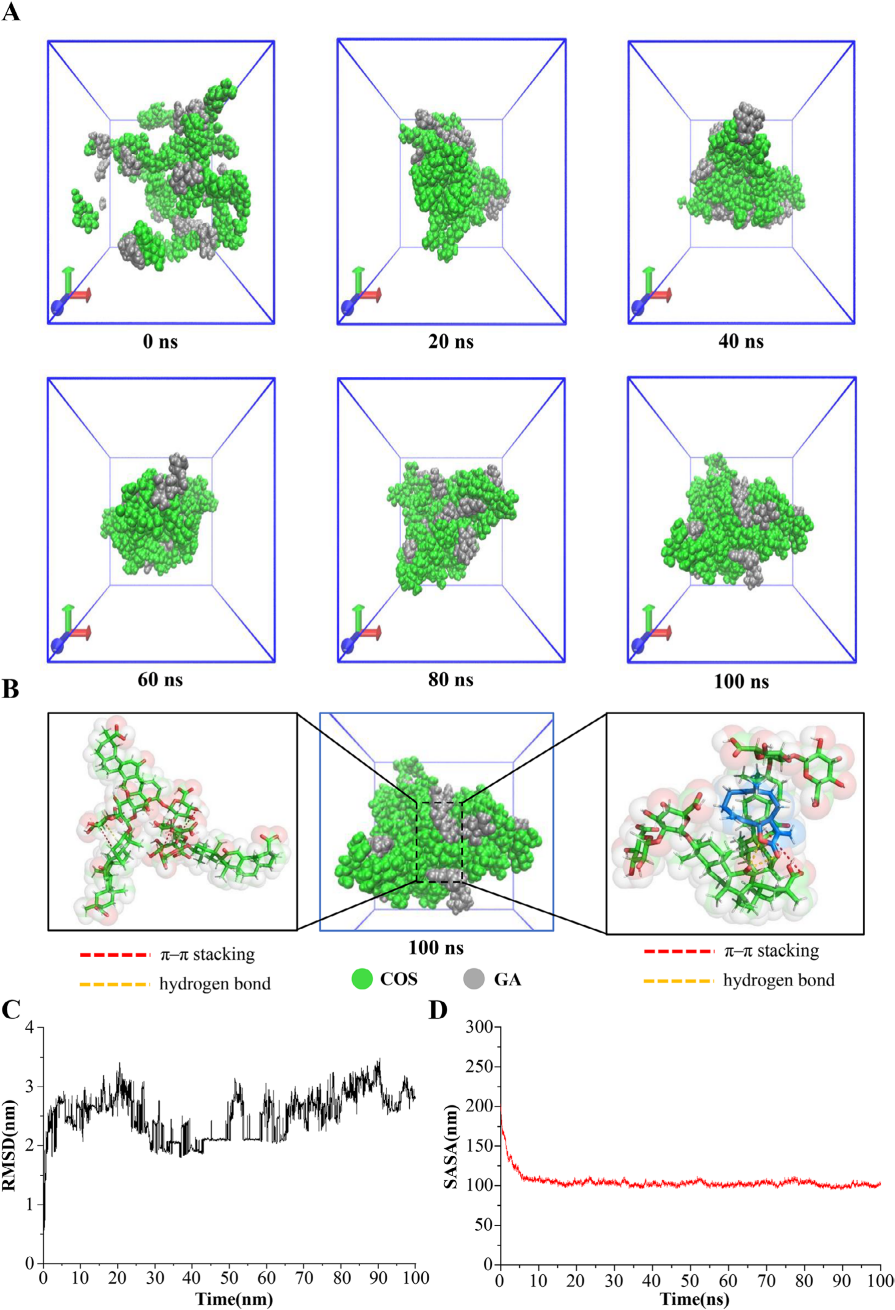
Performing molecular dynamic simulations of COS-GA NPs. (**A**) The structural changes of the COS-GA system were observed at 20 ns intervals during the simulation. (**B**) Interactions at the molecular level within the COS-GA system. (**C**) The Root Mean Square Fluctuation (RMSD) values for the COS-GA system are displayed. (**D**) Analysis of the solvent accessible surface area (SASA) of the COS-GA system

Self-assembly of COS and GA was analyzed using molecular dynamics simulations. The molecular structure that was collected at intervals of 20 nanoseconds was used as the representative conformation in the COS-GA system (Fig. 3A). By the time the simulation reached 20 ns, COS and GA had created stable nanoclusters. The self-assembly was mainly caused by hydrogen bonding and π–π stacking. The size of π–π stacking was measured by LJ-SR in these simulations, and the strength of hydrogen bonding by Coul-SR. The COS-GA system had an average of 36415.88 KJ/mol for LJ-SR and − 324105.27 KJ/mol for Coul-SR. As demonstrated in Figure S1, this suggests that π–π stacking was significantly stronger than hydrogen bonding. The intermolecular forces in the COS-GA system are shown in Fig. 3B. In order to facilitate π–π stacking, GA’s conjugated ring structure allows its hydroxyl group to make hydrogen bonds with COS’s methylenedioxy group. The main form of interaction between GA molecules was π–π stacking. Furthermore, the average RMSD value, which quantifies the magnitude of motion in the molecules, was determined to be 2.53 nm. This finding provides confirmation of the system’s stability during the whole simulation, as depicted in Fig. 3C. To assess the molecular solvent exposure, the SASA was used. A total of 104.81 nm2 of COS-GA surface area was measured. Reduced surface area of solvent-exposed nanoclusters was indicated by a sharp drop in SASA in the initial 0–5 nanoseconds. It then reached a steady state, implying the development of denser and more stable nanoclusters (Fig. 3D). To summarize, COS and GA formed nanospheres primarily through the process of π–π stacking and hydrogen bonding.

### Stability of COS-GA NPs

The stability of COS-GA NPs was assessed under various conditions [17]. As indicated in Table 1, the PDI of COS-GA NPs < 0.5, and the size remained under 1000 nm across all tested conditions, confirming the NPs’ stable nanostate. Variations in ZP closely mirrored the NPs’ stability. Generally, ZP values above ± 30 mV suggest enhanced stability. Although no treatment group achieved the most stable status, the COS-GA NPs remained homogeneous after undergoing centrifugation, heating, cooling, and freeze-thaw cycles, indicating their comparative stability.

**Table 1:** Stability of COS-GA NPs

Treatments	PDI	Size (nm)	ZP (mV)
No Treatment	0.38 ± 0.09	370.72 ± 3.55	13.12 ± 1.05
6000 rpm, 30 min	0.32 ± 0.08	375.36 ± 2.76	11.07 ± 0.75
12,000 rpm, 30 min	0.38 ± 0.05	387.36 ± 1.31	9.22 ± 0.049
50 °C, 30 min	0.32 ± 0.12	413.99 ± 0.89	10.87 ± 0.58
4 °C, 30 min	0.30 ± 0.18	349.06 ± 5.36	9.34 ± 0.19
Freezing and thawing	0.45 ± 0.11	578.59 ± 4.69	9.17 ± 0.12

### Attenuation of DSS-induced mouse UC by COS-GA NPs

**Fig. 4:**
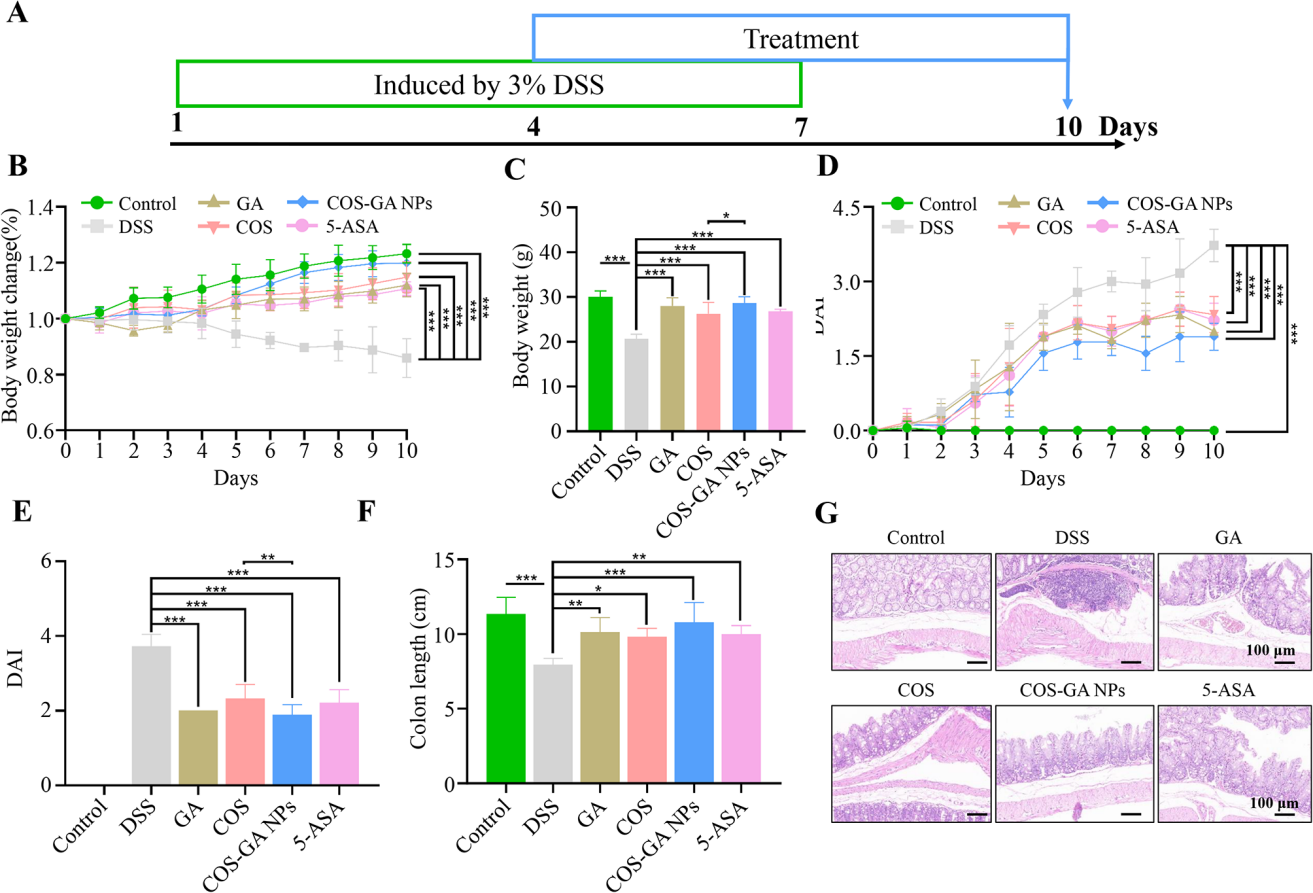
Impact of COS-GA NPs on mice with ulcerative colitis. (**A**) Diagram illustrating the procedure for establishing the model. (**B**) The study examines the fluctuations in body weight over 10 days among several groups, with a sample size of six mice in each group. (**C**) The body weight on day 10 was measured for each group, with a sample size of six mice. (**D**) The DAI score exhibited changes over a period of 10 days. (**E**) The DAI on day 10 was measured for each group. (**F**) The colon lengths were measured in six separate groups. (**G**) The colon slides were stained with H&E. The scale bar indicates a length of 100 μm. Data are mean ± SD, *n* = 6. *P < 0.05, **P < 0.01, and ***P < 0.001

Figure 4A shows the results of the study that evaluated the therapeutic benefits of COS-GA NPs against UC in a mouse model of colitis that was created by DSS. As depicted in Fig. 4B, the Control group of mice showed no change in body weight by day 10, but the DSS-induced colitis group showed a significant decrease. Moreover, on day 10, the DAI was significantly greater in the DSS group than in the Control group, confirming the successful establishment of the UC model (Fig. 4D). Figure 4B and C demonstrate that COS-GA NPs significantly reduced the amount of weight lost due to DSS-induced UC. The DAI score exhibited a notable reduction after a 10-day period in mice that received COS-GA NPs treatment, as shown in Fig. 4D and E. Furthermore, mice that received COS-GA NPs showed enhanced integrity of the colon (Fig. 4F). Although COS and GA alone shown certain therapeutic advantages, the combined use of COS-GA NPs was much more efficacious in mitigating weight loss and ameliorating colon damage. Pathological alterations were additionally assessed using H&E staining. Immune cells penetrating the epithelium completely eliminated the essential gland structure from the colons of the DSS group, as shown in Fig. 4G. Glands treated with COS-GA NPs looked the most normal and well-defined, however all four treatments were effective in restoring gland shape.

### COS-GA NP alleviates UC in mice through anti-inflammatory and antioxidant actions

**Fig. 5:**
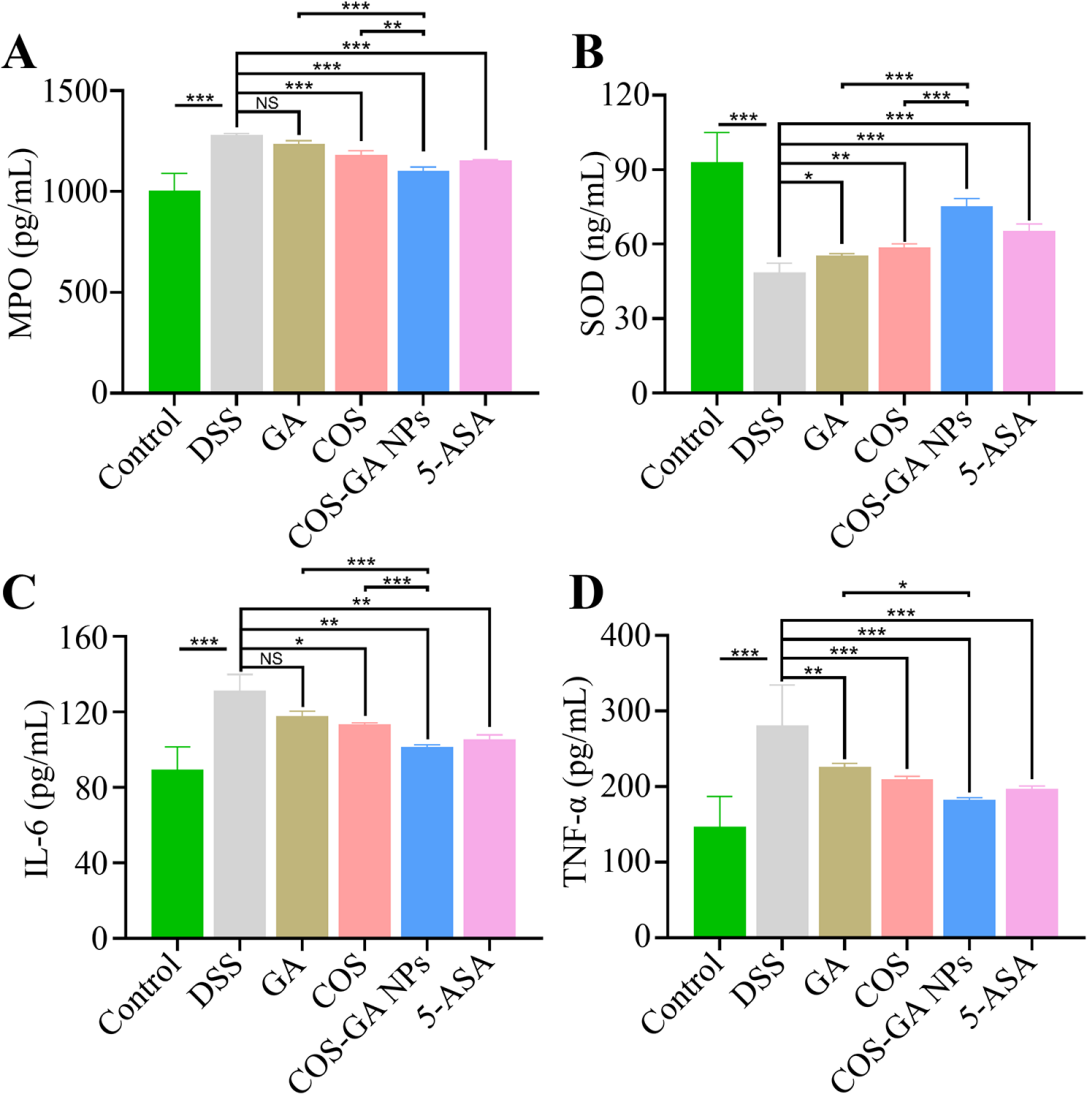
COS-GA NP reduces inflammation and oxidative stress in UC mice. Histograms display changes in IL-1β (**A**), TNF-α (**B**), MPO (**C**), and SOD (**D**) levels in the colon following treatment with COS-GA NP. Data are mean ± SD, *n* = 6. *P < 0.05, **P < 0.01, ***P < 0.001

To assess the anti-inflammatory effects of COS-GA NP in UC-affected mice, IL-1β and TNF-α levels were measured in colon tissues (Fig. 5A and B). DSS treatment elevated IL-1β and TNF-α levels, which COS-GA NP, outperforming COS, GA, and 5-ASA groups significantly reduced. Inflammatory conditions are often linked with oxidative stress; therefore, the effects of COS-GA NP on oxidative stress were evaluated by measuring superoxide dismutase (SOD) and myeloperoxidase (MPO) activities. As shown in Fig. 5C and D, treatment with COS-GA NP markedly increased SOD activity and reduced MPO activity, indicating a reduction in oxidative stress. Notably, COS-GA NP was more effective in enhancing SOD activity and reducing MPO levels compared to other groups.

### Biosafety evaluation of COS-GA NPs

**Fig. 6:**
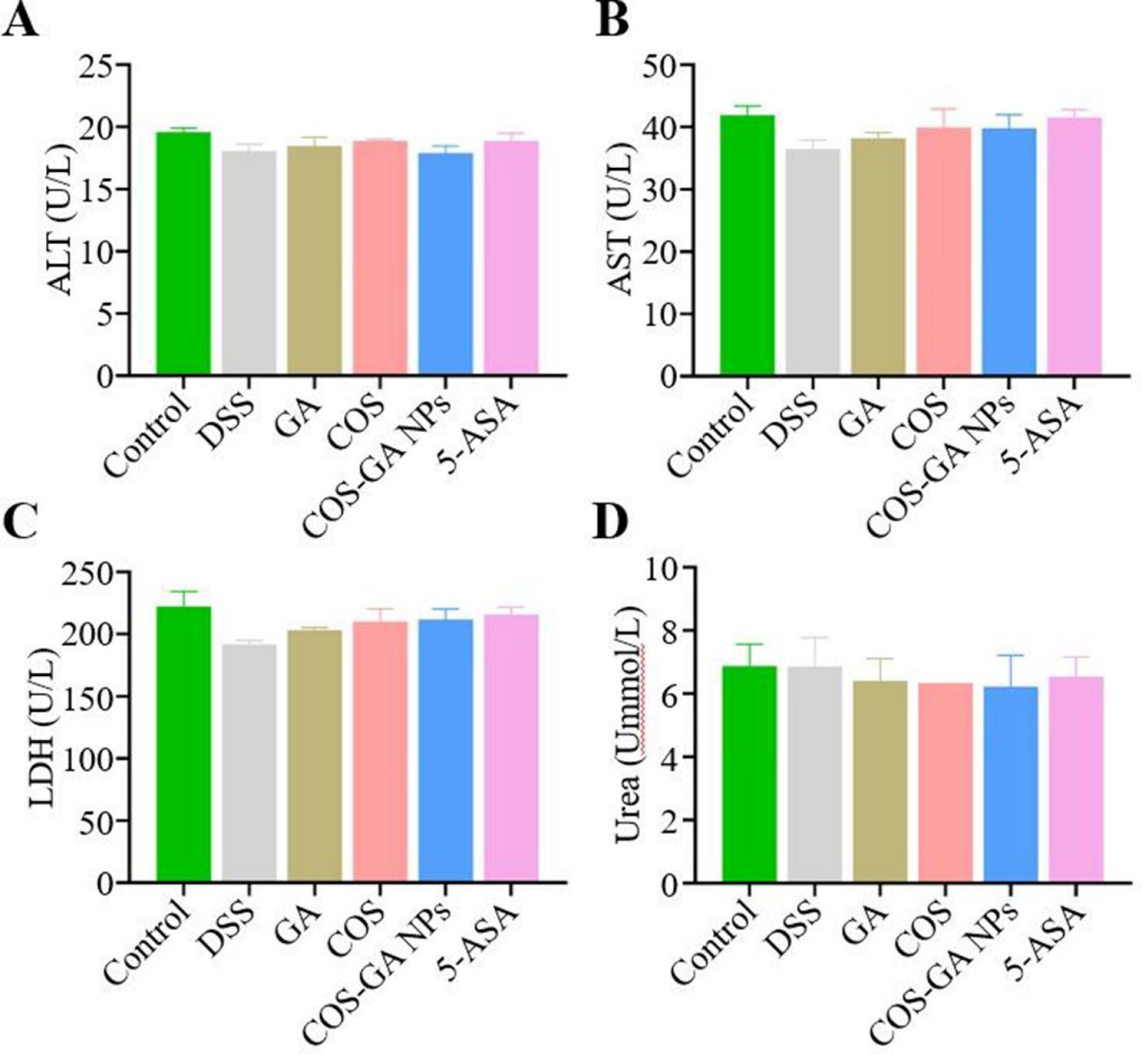
Evaluation of the safety of COS-GA NPs in the management of colitis. Quantification of (**A**) ALT, (**B**) AST, (**C**) LDH, and (**D**) BUN. Data are mean ± SD, *n* = 6

The biocompatibility and biosafety of COS-GA NPs were evaluated in an i*n vivo* study (Fig. 6). Blood urea nitrogen (BUN), alanine aminotransferase (ALT), aspartate aminotransferase (AST), lactate dehydrogenase (LDH), and others were measured in the serum of mice. However, no significant alterations were observed in these markers across all groups. The results validate the safety and compatibility of orally delivered COS-GA NPs, endorsing their potential for use in clinical settings.

## Discussion

The process of self-assembly involves the spontaneous formation of three-dimensional structures or networks by molecules, macromolecules, or composites, which display distinct and original characteristics [17]. It is noted that GA possesses the capability for supramolecular self-assembly, forming distinctive nanostructures [13]. However, literature on the self-assembly of COS and GA is scarce. Thus, we advanced our hypothesis and confirmed the self-assembly process of COS and GA, offering new insights into their mechanisms of action. In this study, we observed that COS and GA self-assemble into nanospheres driven by π-π stacking forces and hydrogen bonding interactions.

NPs, due to their small size, exhibit substantial surface area and surface energy, potentially leading to physical and chemical instability under varying pH, thermal, or gravitational conditions [18, 19]. Therefore, assessing the stability of COS-GA NPs is critical. ZP is an essential stability parameter, representing the electric potential at the slip/shear surface of a nanoparticle in motion within an electric field [20–22]. A higher ZP indicates increased stability, enhancing resistance to degradation and aggregation. Our findings demonstrated that COS-GA NPs maintained stability across diverse environmental conditions. In addition, we found a difference in the particle size of COS-GA NPs in the TEM and Particle size results. This may be due to the differences caused by using different instruments for detection. However, the particle size diameter of COS-GA NPs measured in TEM and Particle size results are consistent with the nanoparticle range.

The combination of COS-GA NPs potentiated the pharmacological effects of COS and GA. The mice in the DSS control group displayed notable symptoms, such as substantial weight loss, bleeding from the rectum, decreased length of the colon, damage to the intestinal lining, and extensive presence of inflammatory cells. These observations corroborate the successful creation of the mouse model for UC [23]. Administration of COS, GA, and COS-GA NPs for a duration of 7 days alleviated the reduction in body weight observed in mice caused with DSS, and maintained the length of the colon. The therapeutic efficacy of COS-GA NPs was shown to be substantially greater than that of COS and GA alone, indicating excellent therapeutic effects against DSS-induced colitis. Increased therapeutic effectiveness of COS-GA is likely due to an increase in the solubility of the drug. What is the deeper mechanism by which COS-GA NPs alleviate UC? Based on the current results, we hypothesize that COS-GA NP is taken orally into the body and reaches the colon via the stomach and small intestine, where it is better absorbed compared to COS and GA due to its smaller particle size, thus acting as an anti-inflammatory and antioxidant, which in turn alleviates UC mice. However, more research is needed on the specific mechanisms.

The results of this study showed that the COS-GA NPs group had a significantly stronger improvement effect on some indicators than the GA and COS groups, which, to some extent, indicates that the therapeutic effect of COS-GA NPs on UC is stronger than that of GA and COS at the same dosage. In addition, the COS-GA NPs showed almost the same therapeutic effect as that of 5-ASA. What’s more, compared with 5-ASA, COS-GA NPs are derived from natural resources, which is more advantageous. Therefore, COS-GA NPs may be promising drugs for the treatment of UC in the future.

In addition, we also evaluated the biocompatibility and biosafety of COS-GA NPs *in vivo.* The results revealed that COS-GA NPs had good biocompatibility and biosafety, which provided a good one for subsequent UC therapeutic studies. Further clinical studies are needed for follow-up.

## Conclusion

The study demonstrated that the natural phytochemicals, COS and GA, self-assemble into nano-spherical particles. The therapeutic potential of COS-GA NPs was much more pronounced than COS and GA. COS-GA NPs demonstrated outstanding therapeutic efficacy in the treatment of colitis induced by DSS by reducing inflammation and oxidative stress. Moreover, it was proven to be safe with the biological systems. On the whole, COS-GA NPs could be a promising use in medication development and colitis management.

### Electronic supplementary material

Below is the link to the electronic supplementary material.

Supplementary Material 1

## Data Availability

The datasets generated and analyzed during the current study are not publicly available due to potential commercial misuse but are available from the corresponding author on reasonable request.

## Abbreviations

COS: Costunolide
DAI: Disease activity index
DSS: dextran sodium sulfate
ELISA: Enzyme-linked immunosorbent assays
H&E: Hematoxylin and Eosin
GA: Glycyrrhizic acid
UC: Ulcerative colitis
TNF-α: tumor necrosis factor-α
NPs: nanoparticles
THF: tetrahydrofuran
