# Supplementary figures and images for: Self-assembled nanoparticles of costunolide and glycyrrhizic acid for enhanced ulcerative colitis treatment
Source: BMC Gastroenterol. 2024 Jul 11;24:223. doi: 10.1186/s12876-024-03313-9 (PMC11241987; doi:10.1186/s12876-024-03313-9)

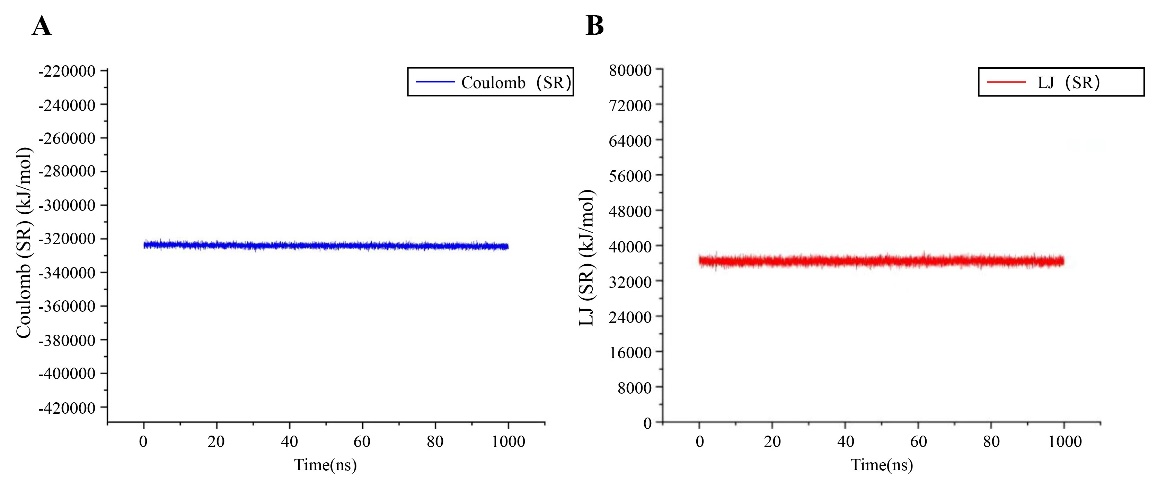


**Figure S1** Coul-SR and LJ-SR in the COS-GA system.

Supplement: Supplementary file 1 — Supplementary Material 1 [file 12876_2024_3313_MOESM1_ESM.docx]
